# Integrated Autofluorescence Characterization of a Modified-Diet Liver Model with Accumulation of Lipids and Oxidative Stress

**DOI:** 10.1155/2014/803491

**Published:** 2014-06-09

**Authors:** Anna Cleta Croce, Andrea Ferrigno, Valeria Maria Piccolini, Eleonora Tarantola, Eleonora Boncompagni, Vittorio Bertone, Gloria Milanesi, Isabel Freitas, Mariapia Vairetti, Giovanni Bottiroli

**Affiliations:** ^1^IGM-CNR, Via Abbiategrasso 207, 27100 Pavia, Italy; ^2^Department of Biology & Biotechnology, University of Pavia, Via Ferrata 9, 27100 Pavia, Italy; ^3^Internal Medicine and Therapeutics, University of Pavia, Via Ferrata 9, 27100 Pavia, Italy

## Abstract

Oxidative stress in fatty livers is mainly generated by impaired mitochondrial ***β***-oxidation, inducing tissue damages and disease progression. Under suitable excitation, light liver endogenous fluorophores can give rise to autofluorescence (AF) emission, the properties of which depend on the organ morphofunctional state. In this work, we characterized the AF properties of a rat liver model of lipid accumulation and oxidative stress, induced by a 1–9-week hypercaloric methionine-choline deficient (MCD) diet administration. The AF analysis (excitation at 366 nm) was performed *in vivo*, via fiber optic probe, or *ex vivo*. The contribution of endogenous fluorophores involved in redox reactions and in tissue organization was estimated through spectral curve fitting analysis, and AF results were validated by means of different histochemical and biochemical assays (lipids, collagen, vitamin A, ROS, peroxidised proteins, and lipid peroxidation -TBARS-, GSH, and ATP). In comparison with the control, AF spectra changes found already at 1 week of MCD diet reflect alterations both in tissue composition and organization (proteins, lipopigments, and vitamin A) and in oxidoreductive pathway engagement (NAD(P)H, flavins), with a subsequent attempt to recover redox homeostasis. These data confirm the AF analysis potential to provide a comprehensive diagnostic information on negative effects of oxidative metabolism alteration.

## 1. Introduction

Oxidative stress results from the imbalance between intracellular generation of reactive oxygen species (ROS) and scavengers, in terms of increased ROS production, decreased antioxidant protection, and failure to repair oxidative damage. The ROS are able to cause structural damages and functional failure of macromolecules containing double bonds, like lipids, proteins, and nucleic acids [1]. Aerobic respiration is the main* in vivo* source of ROS, although fatty acids peroxisomal *β*-oxidation, microsomal cytochrome P450 metabolism of xenobiotic compounds, and stimulation of phagocytosis by pathogens can contribute to their generation. Under normal physiological conditions, a critical balance between ROS generation and neutralization is ensured by the endogenous antioxidant defense systems, including superoxide dismutase (SOD), catalase, or glutathione (GSH) peroxidase activities.

In the liver, ROS are hypergenerated under different altered conditions, such as ischemia, ethanol metabolism in alcoholic hepatitis, and impaired mitochondrial *β*-oxidation. This latter is the dominant oxidative pathway for the degradation of fatty acids under normal physiological conditions and the ROS major source in nonalcoholic fatty liver disorder (NAFLD) [2, 3]. The production of ROS associated with lipid accumulation is a cause of enhanced risk of damage and disease progression to necroinflammation, steatohepatitis, fibrosis, cirrhosis, and eventually hepatocellular carcinoma [4, 5] and of injuries during ischemia/reperfusion of donor organs for transplantation [6, 7].

Because of their very short half-life, ROS at present are measured by means of sophisticated and complex methods such as electron spin resonance or spin trapping procedure or more commonly through their related oxidation end products, such as aldehydes from fatty acid peroxidation (i.e., thiobarbituric acid reacting substances (TBARS)) or immunodetection of carbonyl groups in oxidised proteins. The latter techniques, however, are indirect and time consuming [8].

In this context, autofluorescence optical biopsy (AF-OB) can be expected to provide real-time, supportive diagnostic information on liver altered conditions depending on oxidative stress induction. The AF-OB is based on the AF properties of biological tissues, because of the presence of endogenous fluorophores, and up to now has been applied in clinics mainly for diagnosis in oncology [9]. Endogenous fluorophores under excitation with light at a suitable wavelength give rise to an AF emission, the properties of which depend on nature, amount, physicochemical state, intratissue distribution, and microenvironment of these biomolecules, in a close relationship with morphological and metabolic conditions of the biological substrate [10, 11]. Endogenous fluorophores are thus acting as intrinsic biomarkers, and their AF properties can be exploited as diagnostic parameters for a direct characterization and monitoring of physiological or altered morphofunctional tissue properties, in the absence of perturbation due to exogenous marker administration.

Because of its involvement in several metabolic, biosynthetic, catabolic, and detoxificating functions, the liver contains various kinds of endogenous fluorophores. Among these, the coenzymes NAD(P)H and flavins have been already considered mainly for the AF monitoring of liver functionality in transplantation and surgery, exploiting their emission property dependence on redox state and bound-free condition, in a close relationship with their engagement in energetic metabolism, cell oxidative defense, reductive biosynthesis, and signal transduction [12–20]. Additional endogenous fluorophores are becoming of interest for the early detection of liver disorders and damage, considering the increase in severe liver pathologies and their implications for both disease progression and transplantation [16, 17, 21–30].

Aim of this work is to perform a comprehensive characterization of AF properties of liver tissue undergoing redox metabolism alterations and damage, to improve the AF-OB application in the biomedical research and development of real-time diagnostic procedures. A rat model of liver lipid accumulation and oxidative stress has been obtained through the administration of a hypercaloric methionine-choline deficient (MCD) diet, a validated procedure inducing hepatic steatosis, mitochondrial dysfunction, inflammation, fibrosis, and progression to steatohepatitis [31]. The liver tissue AF properties have been characterized both* in vivo*, via fiber optic probe, and* ex vivo*, with attention to endogenous fluorophores involved in oxidoreductive reactions, namely, pyridinic coenzymes -NAD(P)H- and flavins, or reflecting tissue composition alterations, such as lipids, vitamin A, and lipofuscin like lipopigments, peroxidised proteins, and collagen.

## 2. Materials and Methods

### 2.1. Animal Model

Male Wistar rats were divided into two groups: fed with the MCD diet for 1–4, 8, and 9 weeks, 2 rats per week, and with an isocaloric diet as the control, 3 rats. Rats were anaesthetised (Pentobarbital i.p. injection, 40 mg/kg) and livers exposed to perform* in situ* AF analysis under living conditions, via fiber optic probe. Rats were then sacrificed, liver specimens collected, immediately frozen in liquid nitrogen, and stored at −80°C until being processed for biochemical assays or cut at cryostat (CM1850, Leica Microsystems, GmbH, Wetzlar, Germany). The cryostatic tissue sections were submitted to AF imaging and microspectrofluorometry in the absence of fixation and staining or to histochemical procedures.

The use of the animal model was approved by the Italian Ministry of Health and Pavia University Animal Care Commission.

### 2.2. Spectrofluorometric Analysis

Liver tissue autofluorescence was analyzed* in vivo* by means of an optical multichannel analyzer (Hamamatsu Photonics Deutschland GmbH, Herrsching am Ammersee, Germany; model PMA11) as already described in detail [20]. In brief, excitation light at 366 nm (5.0 W LED, Fraen Corporation, Trivolzio, PV, Italy) was driven to a beam splitting module (Oriel Instruments, Stratford, CT, USA) with a 390 nm dichroic mirror (Chroma Technology Corp, Rockingham, VT, USA). A single optic fiber (300 *μ*m diameter, Fiberlan, Milan, Italy) was used, guiding both excitation and emission light, respectively, to the measuring site and to the detector through the dichroic mirror and a barrier filter (GG 395, Oriel, Newport Corporation, Irvine, CA) coupled with a 17 fiber bundle (200 *μ*m diameter each). The AF signals were collected by inserting gently the fiber optic probe into the tissue. Spectra showing profile alterations indicating deoxyhemoglobin reabsorption were discarded. Each spectral acquisition lasted for 10 sequential scans of 400 ms each (total measuring time of 4 s), and the excitation shutter opened automatically for the collection of the AF signal. Emission spectra were recorded in the 400–750 nm range.

*Ex vivo* AF spectra were collected from cryostatic tissue sections under epi-illumination by means of a microspectrograph (Leitz, Wetzlar, Germany) equipped with an optical multichannel analyzer with a 512-element intensified diode array detector (model 1420/512, EG&G-PAR, Princeton, NJ). The AF signal was excited with a 100 W Hg lamp (Osram, Berlin, Germany) combined with KG1-BG38 antithermal filters. The measurement conditions were 366 nm band-pass interference excitation filter (FWHM 10 nm, T%_366_ = 25) and 390 nm dichroic mirror (T%_366 _< 2), spectra recorded in the 400–680 nm range, for AF; 436 nm band-pass interference excitation filter (FWHM 10 nm, T%_436_ = 40%), TK 450 dichroic mirror (T%_436_ < 2%), 480 nm long-pass filter, and spectra recorded in the 480–750 nm range, for Nile red. Each spectrum acquisition lasted 2 seconds (10 sequential scans of 200 milliseconds each). Unless otherwise reported, spectra were collected with a Leitz 25x objective (NA 0.60) from fixed tissue areas of 4 × 10^4^ *μ*m^2^ selected by a field iris diaphragm.

Curve fitting analysis of AF spectra was performed by means of an iterative nonlinear curve fitting procedure (PeakFit: SPSS Science, Chicago, IL) based on the Marquardt-Levenberg algorithm [32]. The procedures followed for the fitting analysis were already described in detail, as to both the choice of half-Gaussian Modified Gaussian (GMG) spectral function parameters characterizing each single fluorophore and the estimation of their contribution to the AF overall signal [22, 27, 33]. In brief, the spectral functions describing the emission profile of each single fluorophore involved in liver tissue AF were combined by means of subsequent adjustments to match the best fit of the curve representing their sum with the experimental spectrum profile. The spectral parameters of the single fluorophores considered were free NAD(P)H (peak center wavelength, *λ* = 463 nm; FWHM = 115 nm), bound NAD(P)H (*λ* = 444 nm; FWHM = 105 nm), flavins (*λ* = 526 nm; FWHM = 81 nm), vitamin A (*λ* = 488 nm; FWHM = 102 nm), arachidonic acid (fatty acids, *λ* = 470 nm; FWHM = 90 nm), proteins (emission tail, *λ* < 440 nm), and lipofuscin-like lipopigments (*λ* ≈ 587 nm; FWHM = 80 nm). The spectral functions of proteins (viz., collagen) and lipofuscins were allowed to modify for a full spectral combination, because of the variability of their emission profile in dependence of their heterogeneous chemical composition, that is, proteins, lipids, carotenoids, and oxidation and crosslink degree [34].

Spectral peak values were normalized to 100 a.u. before analysis, that was performed through the finding of the true absolute minimum value of the sum of squared deviations (*χ* ^2^), and the goodness of fitting was verified in terms of residual analysis and *r* ^2^ coefficient determination.

### 2.3. Image Analysis

Image analysis was performed by means of an Olympus BX51 fluorescence microscope (Olympus Optical Co. GmBH, Hamburg, Germany) equipped with a 100 W Hg excitation lamp and a 4.1 Mpixel digital photo camera (Olympus Camedia C-4040 zoom). Image acquiring conditions were WU Olympus fluorescence cube (330–385 nm band-pass excitation filter, full width at half intensity maximum of (FWHM) 58 nm, BA420 long-pass barrier filter) for AF; WIB Olympus fluorescence cube (460–490 nm band-pass excitation filter, FWHM 16 nm, and BA515 long-pass barrier filter) for Nile red fluorescence; Olympus 20x UplanFl objective (NA 0.50). For AF studies, microscope excitation setup was adjusted to obtain a light fluence rate quite comparable to that used for microspectrofluorometric analysis (6.35 mJ cm^−2^ per second).

Tissue sections submitted to histochemical specific analyses were observed under bright field light, unless otherwise reported.

### 2.4. Histochemistry

Vitamin A was visualized with a gold chloride method [35]. Frozen sections were first fixed with 2% glutaraldehyde in cacodylate buffer 0.1 M (pH 7.4) for 20 min at 4°C and hence with 0.2% OsO_4_ in cacodylate buffer 0.1 M (pH 4.2) for 2 min in the dark, to be then stained with a AuCl_3_ solution (1 mL of 1% AuCl_3_, 1 mL of 0.1 N HCl, and 98 mL of H_2_O) overnight at 20°C and postfixed with 1% OsO_4_ in cacodylate buffer 0.1 M (pH 4.2) for 1 h in the dark. The gold precipitates were visualized under dark field light microscopy.

Visualization of ROS was performed on unfixed frozen sections with the DAB-Mn^2+^-Co^2+^ histochemical reaction. The presence of cobalt ions in the medium gives then a dark blue product [36].

Lipid detection was performed by means of Nile red (Eastman Kodak Co., Rochester, NY, USA) fluorochromization [37]. After AF image acquisition, 25 *μ*m thick sections were gently covered with a drop of Nile red dissolved in glycerol (1.25 *μ*g/mL), mounted with coverslips, and submitted to imaging or microspectrofluorometric analysis 5 min later.

Protein peroxidation was detected in terms of protein carbonyls using a modified protocol from Frank et al. [38]. Frozen tissue sections (6 *μ*m thick) were fixed using a mixture of diethyl ether and ethanol (1 : 1, v/v) for 15 min, before being incubated in 0.3% of 2,4-DNPH dissolved in absolute ethanol containing 1.5% (v/v) sulfuric acid for 16 h, washed in absolute ethanol containing 1.5% (v/v) sulfuric acid for 5 min, and then rehydrated. Endogenous peroxidase activity was blocked with a solution of 3% H_2_O_2_ in 10% methanol for 20 min. Tissue sections were then processed for specific immunolabeling, by means of a specific DNPH-protein carbonyl-adduct-specific rabbit antibody, followed by peroxidase-conjugated secondary anti-rabbit antibody (ImPRESS reagent kit, Vector, Burlingame, CA, USA) and 3,3-diaminobenzidine chromogen system (Dako Italia S.p.A., Milano, Italy). The slides were mounted with glycerol gelatin for observation at microscope. All operations were performed at room temperature.

Collagen fiber detection was performed by means of Sirius red (Direct Red 80) staining [39]. Tissue sections, unfixed and air-dried for at least 48 h, were immersed in the staining solution (0.1% of Sirius red in picric acid-saturated aqueous medium, 1 h) and washed in two changes of acidified water (0.5% acetic acid).

For AF imaging and for each kind of histochemical procedure, images shown in the respective figures were chosen as representatives of the patterns obtained from each of the control or MCD diet administered rats (6 tissue sections per rat).

### 2.5. Biochemical Assays

Tissue ATP was measured by the luciferin-luciferase method with the ATP Bioluminescence Assay Kit CLS II (Roche Molecular Biochemicals, Milan, Italy). The hepatic concentration of total glutathione (GSH) was measured by an enzymatic method (Cayman Chemical Co., Ann Arbor, MI, USA). The extent of liver lipid peroxidation in terms of thiobarbituric acid reactive substances (TBARS) formation was measured according to the method of Esterbauer and Cheeseman [40]. The TBARS concentrations were calculated using malondialdehyde (MDA) as standard. Protein content was assayed by the method of Lowry et al. [41].

Unless otherwise stated, chemicals were purchased from Sigma-Aldrich, Buchs, Switzerland.

### 2.6. Statistical Analysis

The MedCalc Statistical Software (version 13.1.2; MedCalc Software bvba, Ostend, Belgium) was used for the comparison among groups of data from control and MCD diet fed rats, with reference to the single endogenous fluorophores contributing to the AF overall signals recorded* in vivo* or* ex vivo* or to biochemical components.

The differences were evaluated by means of one way analysis of variance (ANOVA). The analysis of the normality of data distribution was followed by a Student-Newman-Keuls test for all pairwise comparisons in the case of normally distributed data groups or by the nonparametric Kruskal-Wallis test and post hoc analysis for the groups including nonnormally distributed data. The value of *P* < 0.05 was considered to indicate statistical significance.

## 3. Results

### 3.1. *In Vivo* and* Ex Vivo* Spectrofluorometry

**Figure 1:**
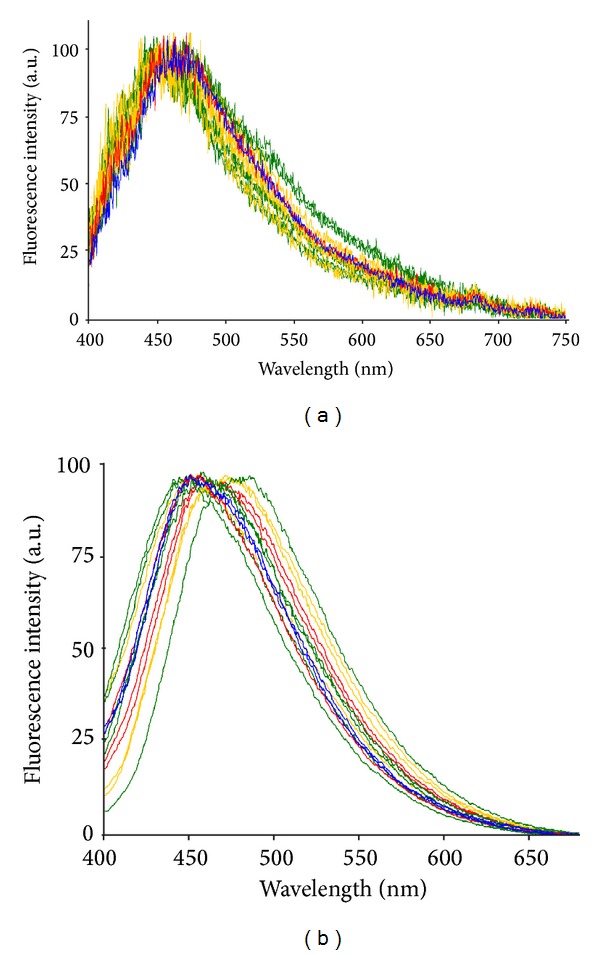
Examples of AF spectra recorded* in vivo* (a), via fiber optic probe, or* ex vivo* (b) via microspectrofluorometry, showing the range of changes in the emission profile induced by MCD diet administration (1 week—red; 2-3 weeks—yellow; 4–9 weeks—green) in comparison with the control (blue). Spectra were normalized to the peak maximum for presentation.

The AF spectra collected* in vivo*, via fiber optic probe directly from liver tissue, showed changes in the emission profile between control and MCD diet rats and among rats administered with MCD diet for increasing times (Figure 1(a)). Spectra were normalized to the peak maximum values for an easier comparison of the emission profile. With respect to the control, the spectra recorded after 1 MCD diet week showed a slight widening towards the blue region. The variability in the spectral shape increased at 2-3 MCD diet weeks, in terms of narrowing or widening in the 400–470, 470–700 nm regions, and was even more marked at 4–9 MCD diet weeks.

Comparable results were provided by* ex vivo* microspectrofluorometric analysis on tissue sections, although with an apparent higher variability in both peak position and spectral profiles (Figure 1(b)).

### 3.2. Curve Fitting Analysis

A curve fitting analysis was performed on both* in vivo* and* ex vivo* AF spectra, to estimate the relative contribution of the main endogenous fluorophores to the whole liver tissue emission and their changes depending on the MCD diet administration time.

**Figure 2:**
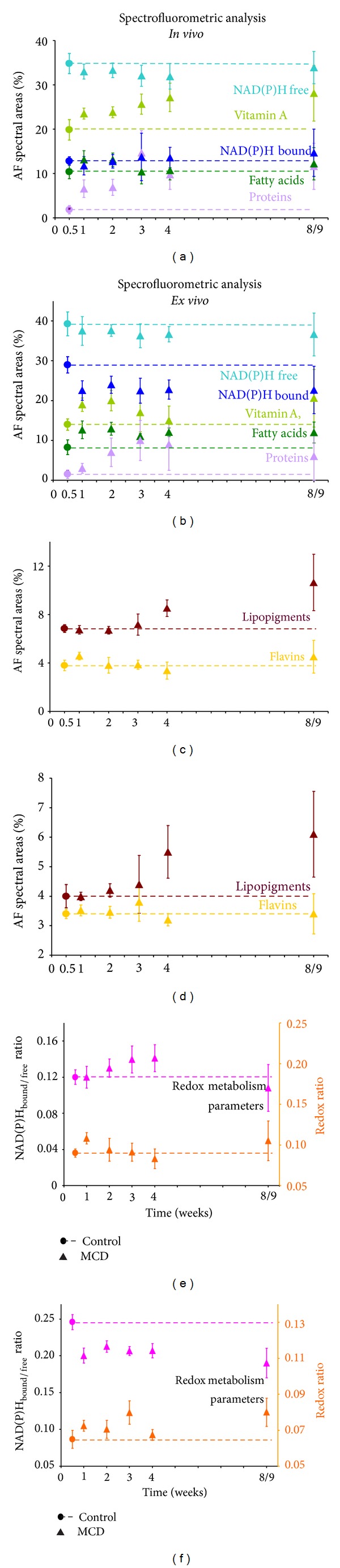
Curve fitting analysis: estimation of the relative contribution of the different endogenous fluorophores to the whole liver AF emission collected* in vivo* (a, c) and* ex vivo* (b, d) in control and MCD diet administered rats; NAD(P)H_bound/free_ and redox ratios calculated from* in vivo* (e) and* ex vivo* (f) data. The results are presented as mean values ± S.D. (*in vivo* analysis: 5 measurements per rat;* ex vivo* analysis: 5 measurements, per 3 liver tissue sections, per rat). Statistical analysis: preliminary inspection showed that all data within each group were normally distributed, except for NAD(P)H_bound_,* in vivo* data.* In vivo* significant differences: panel a: proteins, among control and all MCD diet week groups, except for MCD diet 3, 4 versus 8/9 weeks; fatty acids, control versus MCD diet 1, 2, and 8/9 weeks, MCD diet 1, 2 versus 3, 4 weeks, and MCD diet 3, 4 versus 8/9 weeks; vitamin A, among control and all MCD diet week groups, except for MCD diet 1 versus 2 weeks, and MCD diet 4 versus 8/9 weeks; NAD(P)H_bound_, MCD diet 1 versus 3, 4, and 8/9 weeks; NAD(P)H_free_, control versus MCD diet 1–4 weeks. Panel c: lipopigments control and MCD diet 1–3 versus 4, 8/9 weeks; flavins, control versus MCD diet 1, 8/9 weeks, MCD diet 1 versus 2–4 weeks, and MCD diet 2–4 versus 8/9 weeks. Panel e: NAD(P)H_bound/free_ ratio, control and MCD diet 1, 2 versus 3-4 weeks, MCD diet 3, 4 versus 8/9 weeks; redox ratio, control and MCD diet 2–4 versus 1, 8/9 weeks.* Ex vivo* significant differences: panel b: proteins, control versus all MCD diet week groups, MCD diet 1 versus 2–8/9 weeks, and MCD diet 3 versus 2–8/9 weeks; fatty acids, control versus all MCD diet week groups and MCD diet 3 versus 1, 2, 4, and 8/9 weeks; vitamin A, among control and MCD diet 1–3, 8/9 weeks and MCD 4 versus 1–3, 8/9 weeks; NAD(P)H_bound_, control versus all MCD diet week groups; NAD(P)H_free_, control versus MCD diet 2–8/9 weeks. Panel d: lipopigments control and MCD diet 1–3 versus 4,8/9 weeks; flavins, control and MCD diet 1, 2, 4, and 8/9 versus 3 weeks. Panel f: NAD(P)H_bound/free_ ratio, control versus all MCD diet week groups and MCD diet 1 versus 2, 8/9 weeks; redox ratio, control versus MCD diet 1–3, 8/9 weeks and MCD diet 1,2,4 versus 3,8/9 weeks. Before NAD(P)H_bound/free_ and redox ratio calculation, the values of NAD(P)H in the bound form were corrected for the AF emission yield (3 : 1 NAD(P)H_bound_ versus NAD(P)H_free_) [12]. Redox ratios were calculated as (flavins)/(NAD(P)H_total_ + flavins) [42].

Figures 2(a)–2(d) show that in general the levels of vitamin A, proteins, and lipopigments increase in MCD diet livers in comparison with the controls, while smaller changes can be observed for the fatty acid component (Figures 2(a) and 2(b)). With respect to* in vivo* data,* ex vivo* analysis revealed some differences concerning vitamin A and fatty acids, becoming more appreciable after 3 weeks of MCD diet administration. As to the AF biomarkers of oxidative metabolic pathways,* in vivo* data indicated a slight, continuous increase or decrease for the respective bound and free forms of NAD(P)H. These changes were well evidenced by the rising of the NAD(P)H_bound/free_ ratio, becoming significantly higher than the control at 3-4 weeks of MCD diet (Figure 2(e)). It is interesting to note that the estimation of the relative contribution of NAD(P)H in tissue sections provided values generally of a greater extent as compared to* in vivo* data. The MCD diet samples, in particular, showed that the NAD(P)H in the bound form was almost doubled in* ex vivo* as compared to* in vivo* measurements. As a consequence, the NAD(P)H_bound/free_ ratio was much higher in* ex vivo* than* in vivo* conditions. Differences were observed also in the time course of the NAD(P)H_bound/free_ ratio;* in vivo* measurements showed increasing values from the control to the 4th week of MCD diet (Figure 2(e)), while* ex vivo* measurements evidenced a ratio value that was always significantly lower in MCD diet samples than in the control (Figure 2(f)). As to the redox ratio, a value higher than the control was evidenced* in vivo* after 1 MCD diet week and after 3 and 8/9 MCD diet weeks in* ex vivo* samples (Figures 2(c) and 2(f)).

The changes in liver tissue biochemical composition indicated by AF spectrofluorometric analysis were verified by means of subsequent AF imaging, histochemical, and biochemical assays.

### 3.3. Autofluorescence Imaging

**Figure 3:**
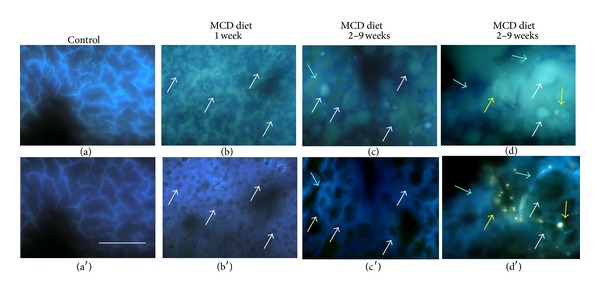
Examples of AF distribution patterns from unfixed, unstained liver tissue sections from control (a) and MCD diet administered rats (b–d), at *t* = 0 and after 10 sec of continuous exposure to excitation light. Irradiation results in the persistence of the bright fibrous network in control (a′). In MCD diet samples (b′–d′), excitation light results in the disappearance of the greenish signal rising from the vesicles (white arrows), in parallel with the persistence of fibrous structures (light blue arrows). Bright granules are also observable, in particular at 9 weeks of MCD diet administration, ascribable to lipofuscin like material (yellow arrows) due to their yellowish emission color and resistance to irradiation. Bar: 160 *μ*m.

The AF imaging confirmed the increasing in the etherogeneity of liver tissue following MCD diet administration. The AF patterns of the control allowed recognizing the anastomosing plates of bluish hepatocytes, surrounded by a brighter emitting network, consistently with the typical arrangement of extracellular reticulin fibers and the high emission efficiency of connective proteins (Figure 3(a)). In MCD diet samples, AF images evidence numerous vesicular structures, much brighter than the surrounding parenchyma, widely spread and localized both inside and around hepatocytes after 1 MCD diet week, and showing markedly increased dimensions at longer MCD diet administration times (Figures 3(b)–3(d)). The continuous irradiation with excitation light resulted in a slight fading of the AF signal in the areas ascribable to hepatocytes in the control (Figure 3(a′), in parallel with a considerable decrease in the vesicle emission and with a persistence of lightly blue structures in MCD diet samples (Figures 3(b′)–3(d′)). The latter phenomenon was more evident from the second week of MCD diet, consistently with the photophysical properties of photoresistant extracellular fibrous proteins, namely, collagen (Figures 3(b′)–3(d′)). The fading of the vesicle signal can be explained with a prevailing presence of the photosensitive vitamin A and of a lipid fraction characterized by marked AF emission and photolability. The presence of different kinds of lipids and changes in their relative fractions between the control and MCD diet samples is supported by the results from Nile red fluorochromization described in the next section.

### 3.4. Histochemical Analysis

**Figure 4:**
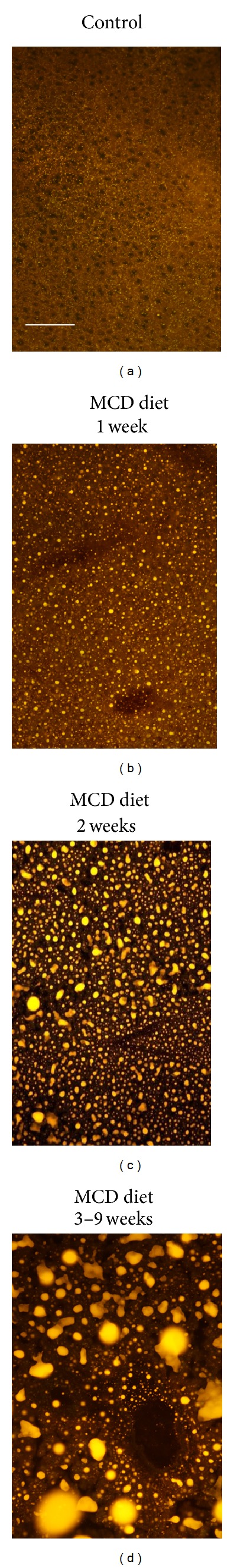
Examples of distribution patterns of lipids, evidenced as bright yellow fluorescing vesicles after Nile red fluorochromization of cryostatic, unfixed sections of liver tissue from control (a) and MCD diet administered rats (b–d). Bar: 200 *μ*m.

Nile red fluorochromization revealed lipid localization as gold-yellow bright emitting droplets and vesicles of variable dimensions, within the orange hepatocyte parenchyma. In the control, small and faint droplets were mainly lining the hepatocyte margins (Figure 4(a)). A similar distribution pattern occurred also at 1 week of MCD diet, although in this case the vesicles were brighter, more numerous, and unevenly distributed in the tissue. The dimension and brightness of droplets and vesicles increased dramatically after 2 weeks of MCD diet (Figures 4(b)–4(d)).

**Figure 5:**
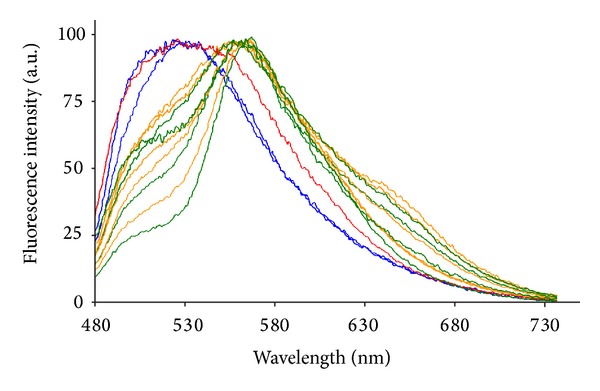
Examples of fluorescence emission spectra collected from liver cryostatic tissue sections submitted to Nile red fluorochromization. Control (blue), MCD diet administration: 1 week (red), 2-3 weeks (yellow), and 4–9 weeks (green). Spectra are normalized to the peak maximum for presentation.

The increase in the amount of lipids was accompanied by changes in their nature, as evidenced by the Nile red spectral analysis. The Nile red emission recorded from the fluorochromized tissue sections showed emission bands in the 480–550 nm, 540–600 nm, and 600–690 nm spectral regions, ascribable, respectively, to a tail of natural fluorescence, apolar lipids, that is, triglycerides, and polar lipids, that is, phospholipids. The 480–550 nm band was predominant in the control, while the 540–600 nm band became appreciable after 1 MCD diet week, to become more evident and accompanied by the 600–690 nm band after 2 MCD diet weeks (Figure 5).

**Figure 6:**
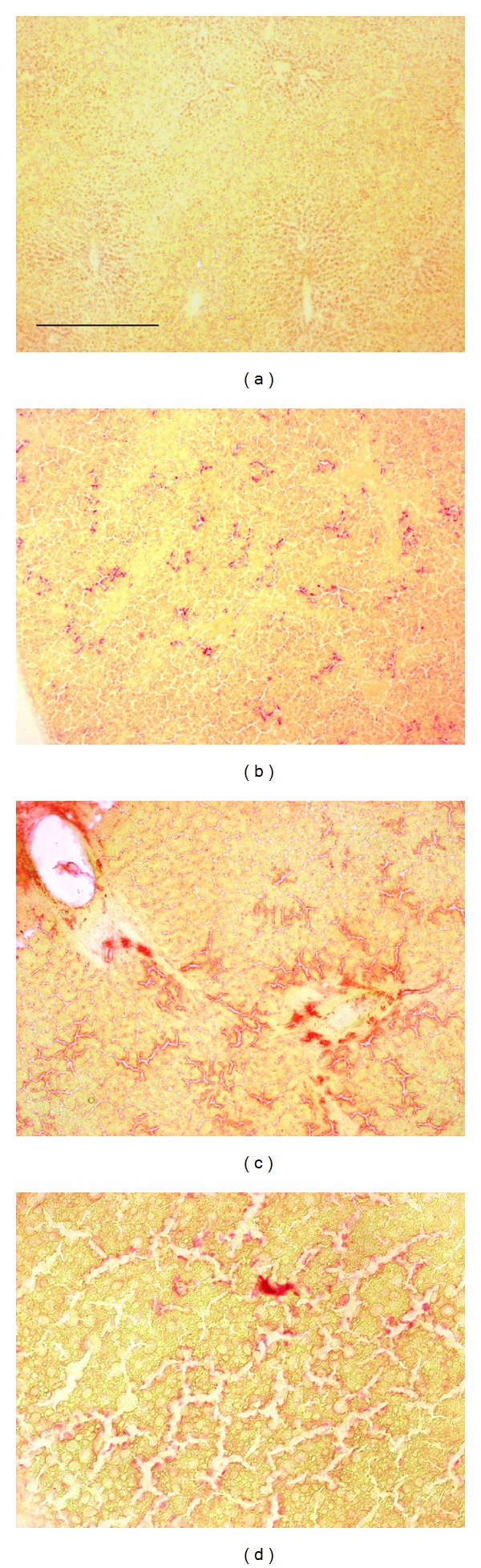
Examples of patterns from cryostatic liver tissue sections submitted to Sirius red staining, evidencing collagen as red fibrous structures. Control (a); MCD diet, 2 weeks (b), 3 weeks (c), and 8/9 weeks (d). Bars: 1000 *μ*m (a, b), 400 *μ*m (c), and 200 *μ*m (d).

Sirius red staining revealed the presence of collagen as red fibrous structures within the yellowish parenchyma. Fibrous structures were detectable already after 2 weeks of MCD diet and delineated micronodules in the parenchyma. Porto-central fibrosis was then becoming appreciable, persisting up to 4 MCD diet weeks, while at 8-9 weeks the tissue was much more disorganized, showing remnants of disorganized fibrous structures along with some vesicles, that is, lipid droplets (Figure 6).

**Figure 7:**
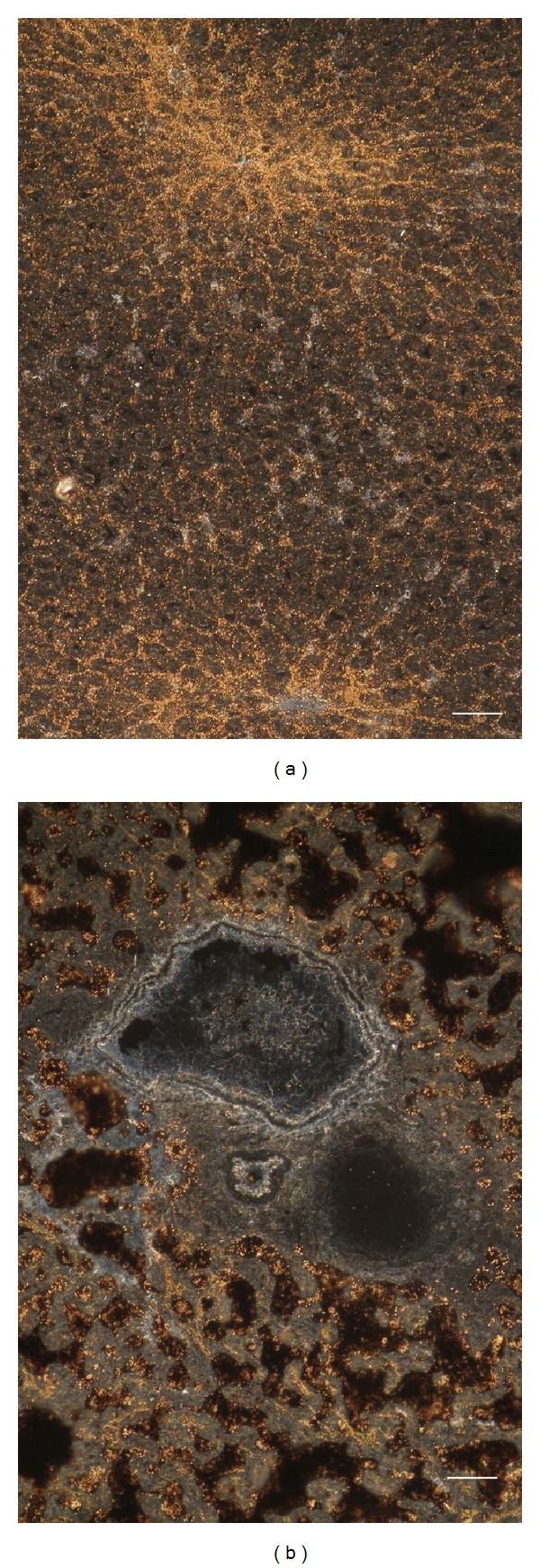
Examples of intratissue distribution patterns of vitamin A, evidenced in terms of yellow reflection of metallic gold precipitate by means of dark-field microscopy. The gold precipitate is present along the sinusoids, within presumed stellate cells and in the cytoplasm of hepatocytes in control (a), or at the periphery of large lipid droplets and in hepatocyte bile canaliculi in MCD diet, 2 weeks (b). In (b), a whitish reflection from the connective tissue allows identifying blood vessels and the bile duct in the portal region. Bar: 50 *μ*m.

The gold chloride reaction for visualizing vitamin A is based on the reduction of gold chloride to metallic gold by retinol molecules, giving a black precipitate observable under bright-field microscopy [35]. Since unsaturated lipids can be also stained in brown by OsO_4_, giving a poor contrast to the specific precipitate due to vitamin A, this drawback was overcome by using dark-field microscopy, as only metallic gold reflects the illumination light. In the control, the gold reflection was present mainly in sinusoidal cells of the periportal region, which are presumed to be vitamin A storing, stellate cells, and in the cytoplasm of hepatocytes (Figure 7(a)). Following MCD diet administration, cytoplasm of hepatocytes was distorted by large lipid droplets, where dark-field microscopy allowed easily discriminating the reflection of gold deposed due to vitamin A (Figure 7(b)).

**Figure 8:**
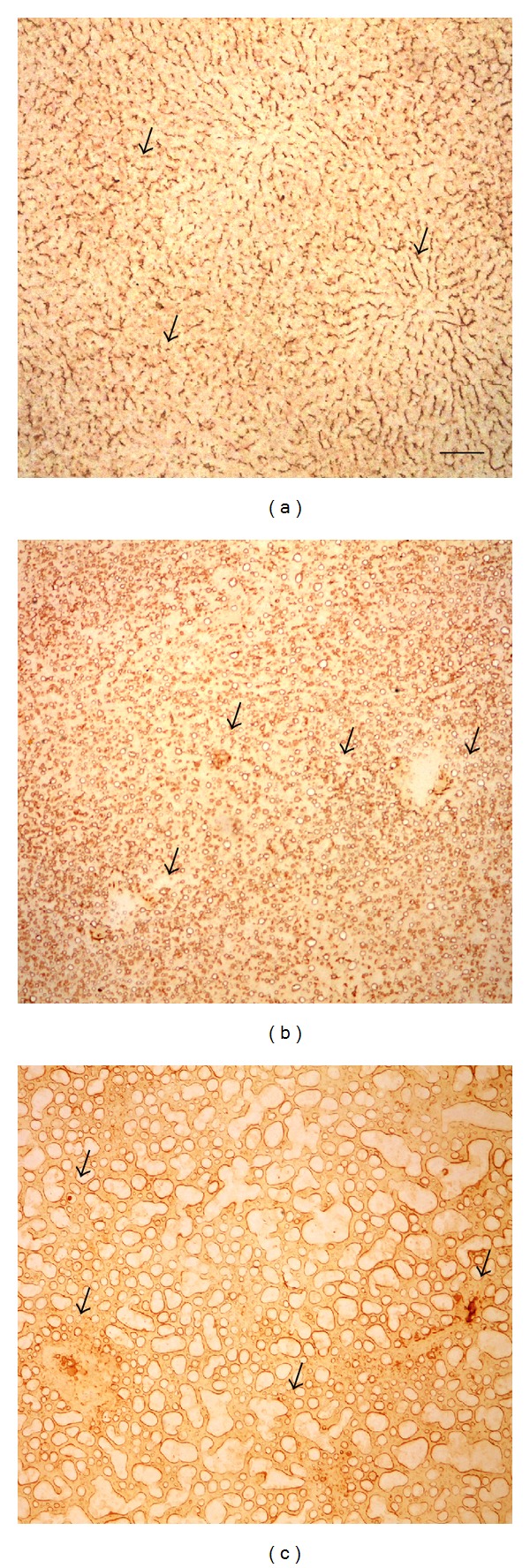
Examples of intratissue distribution patterns of peroxidised proteins, evidenced as the brownish product of immunolabeling. Immunopositivity is detectable along the perisinusoidal domain of the hepatocyte membranes in the control (a, arrows), around lipid vacuoles in MCD diet, 1, 2 weeks (b, c arrows). Bar: 100 *μ*m.

The positive labeling for the peroxidised proteins in the control was mainly detectable along the perisinusoidal domain of the hepatocyte membrane (Figure 8(a)), while after 1 and 2 MCD diet weeks it was observable around lipid vacuoles. At times longer than 2 MDC diet weeks, some aggregates of positive material were clearly detectable also in periportal regions (Figures 8(b) and 8(c)). Peroxidated protein distribution in MCD treated rat liver was consistent with the structural features of lipid droplets, surrounded by a phospholipid monolayer with numerous proteins decorating their surface [43].

**Figure 9:**
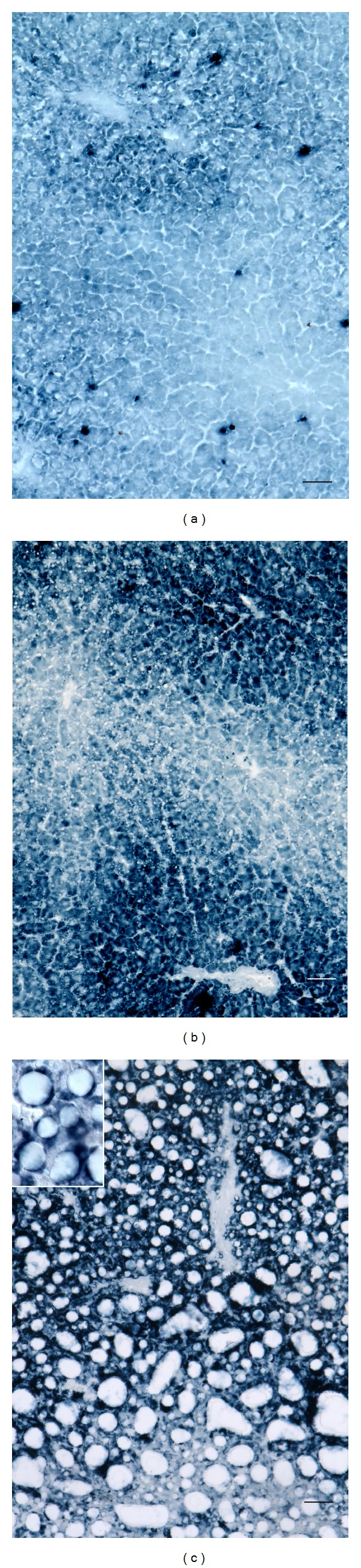
Examples of intratissue distribution patterns of ROS, evidenced as blue precipitate. The staining is moderately intense in the perisinusoidal region of periportal hepatocytes and very marked in sinusoidal cells scattered throughout the lobule, likely Kupffer cells, control (a), and much more appreciable in the cytoplasm of hepatocytes in the periportal and midzone regions in MCD diet, 1 week (b), or in the regions surrounding lipid droplets in steatotic hepatocytes, MCD diet, 2 weeks (c). Bar: 50 *μ*m.

The presence of ROS was demonstrated as a dark blue precipitate [36]. In the control, this precipitate was moderately intense in the perisinusoidal region of the cytoplasm of hepatocytes in the periportal regions, where oxidative reactions are more intense than the centrilobular ones, while a strong reaction was observed in sinusoidal cells scattered in the parenchyma, likely Kupffer cells (Figure 9(a)). In fact, in the liver under normal conditions, ROS are produced mainly as a by-product of aerobic respiration in hepatocytes mitochondria and in the plasma membrane of phagocytosing Kupffer cells. After 1 week of MCD diet administration, a more marked ROS presence was found, in particular in the cytoplasm of hepatocytes in the periportal and midzonal regions of the lobule (Figure 9(b)). After 2 weeks of MCD diet administration, the ROS reaction was further enhanced in the cytoplasm of hepatocytes surrounding lipid droplets, in particular in the periportal region (Figure 9(c)).

### 3.5. Biochemical Assays

**Figure 10:**
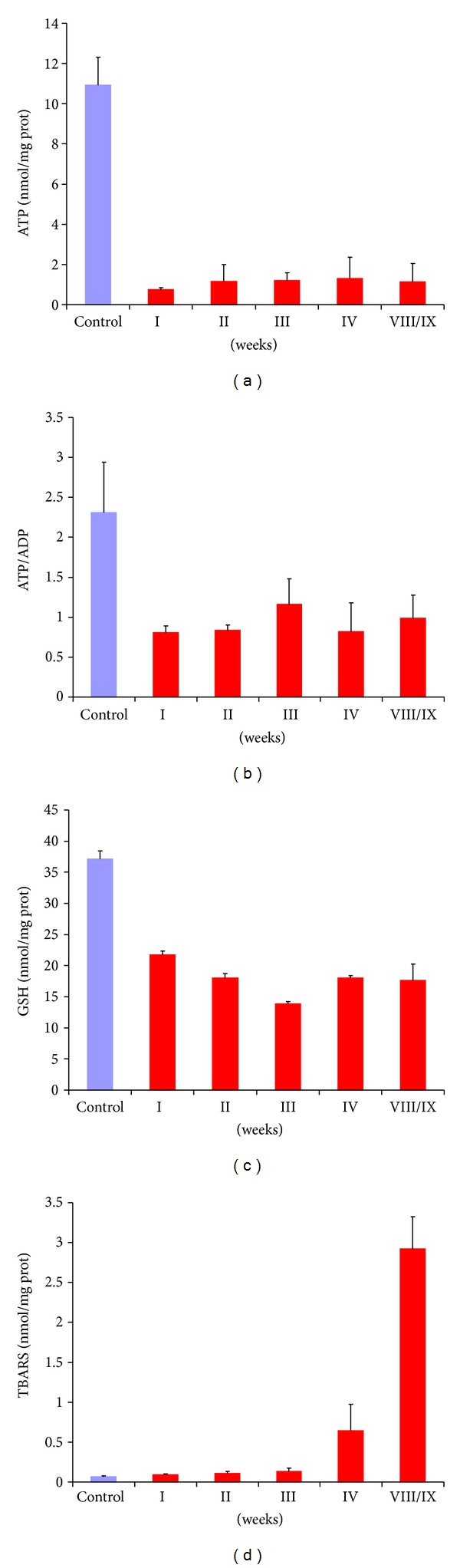
ATP levels (a), ATP/ADP ratio (b), GSH content (c), and TBARS formation (d) in the liver tissue in the control and after MCD diet administration. Data obtained from assays performed in triplicate for each of control or MCD diet rat are presented as means ± S.E.M. Statistical analysis: preliminary inspection showed that all data within each group were nonnormally distributed. Significant differences: panel a: control versus all MCD diet groups; panel b: control versus all MCD diet groups, MCD diet 3 versus 1,2, and 4 weeks; panel c: control versus all MCD diet groups, MCD diet 1 versus 2–8/9 weeks, and MCD diet 3 versus 1, 2, 4, and 8/9 weeks; panel d: control versus all MCD diet groups, MCD diet 1–3 versus 4, 8/9 weeks.

Biochemical assay performed on tissue homogenates demonstrated a very marked decrease in the intratissue amount of ATP content, ATP/ADP ratio, and GSH already after 1 MCD diet week with respect to control rats (Figures 10(a)–10(c)). On the contrary, liver TBARS were slightly rising during the first 3 weeks of MCD diet to become much higher at longer times (Figure 10(d)).

## 4. Discussion

The administration of the MCD diet resulted in the expected rapid alteration of rat liver tissue properties, consistently with the literature describing its ability to induce liver tissue alterations and disease progression [31]. In fact, lipid accumulation and the rising of negative effects of oxidative stress during MCD diet administration are confirmed by different histochemical specific staining and biochemical assays. Both changes in biochemical composition and structural organization of liver parenchyma can affect AF emission properties, in terms of signal distribution patterns and spectral profiles.

Information on the changes in the relative presence of the various endogenous fluorophores is provided in particular by curve fitting analysis procedures, similarly to an* in situ* biochemical analysis. The results from AF spectra obtained* in vivo* and* ex vivo* are comparable concerning the altered presence of vitamin A, proteins, namely, collagen, lipopigments, and lipids in the MCD diet livers in comparison with the control. The changes in vitamin A, collagen, and lipopigments, in turn, are consistent with AF imaging and the response to the continuous irradiation with the excitation light. The rapid fading of the signal rising from the vesicles or the persistence of bluish structures is ascribable to the respective presence of the photolabile vitamin A and of the photoresistant collagen and lipopigments, as supported by specific histochemical staining [27, 43–46]. Some discrepancies between* in vivo* and* ex vivo* measurements can be explained by the increase in tissue heterogeneity during MCD diet administration, entailing an enhanced variability in the level of each fluorophore contribution to the whole tissue AF emission. In particular, the variability in spectral peak position and profile shown by* ex vivo* data as compared to the* in vivo* ones can be ascribed to more selective detection of fluorophores predominating in each measured field. As to the lipids, spectrofluorometric AF data collected both* in vivo* and* ex vivo* do not show the extent of changes that could be expected from the AF imaging and Nile red fluorochromization data, indicating both an increase in their amount and changes in fractions with different polarity degree [37]. In this respect, it is to take into account that only a part of lipids can give rise to a noticeable AF emission, obviously depending on lipid molecular features. Preliminary indications demonstrated that marked AF signals and photolability are exhibited by fatty acids such as arachidonic, linoleic, and stearic acids, while other derivatives such as butirric and palmitic acids, triglycerides, and phospholipids are in general poorly fluorescent ([22, 24, 47] and unpublished results). Further and complex studies would be however required to define more precisely the AF properties of the numerous lipid derivatives. The negative effects induced by oxidative stress and oxidative damages are supported by the histochemical detection of the increase in the presence of ROS and oxidised proteins and by the tissue concentration of TBARS [48]. The AF biomarkers of oxidative metabolic pathways, the coenzymes NAD(P)H and flavins, exhibited some differences between* in vivo* and* ex vivo* evaluation condition.* In vivo* data showed a slight rising in the NAD(P)H_bound/free_ ratio from the second to the fourth week of MCD diet, while a noticeable increase was demonstrated for the redox ratio already at the first MCD diet week, to return to control levels at later time points.

These results suggest a fast rising in the tissue oxidation, in agreement with the more marked presence of ROS detected by histochemistry at the first MCD diet week. This observation is consistent with the very rapid alteration of antioxidant and oxidative respiration functions, demonstrated by the drop of intratissue ATP and GSH levels already at the first MCD diet week. In contrast with histochemical and biochemical data, however, the* in vivo* AF analysis is able to indicate an attempt of liver tissue to counteract oxidative stress. Anaerobic and antioxidant pathways seem to be engaged to recover tissue redox homeostasis at longer MCD diet times, despite the increase in the ROS accompanying the increase in lipid vesicles. As compared to* in vivo*,* ex vivo* analysis reveals higher NAD(P)H contribution values, in particular for NAD(P)H in the bound form, becoming almost doubled. This apparent discrepancy can be explained with the discontinued oxygenation at the collection of tissue sample, resulting in the accumulation of NAD(P)H at its binding sites. Interestingly, while the time course of the contribution of free NAD(P)H is similar between* in vivo* and* ex vivo* measurements,* ex vivo* data evidence a significant difference between the control and MCD diet samples as to the contribution of bound NAD(P)H, likely to be ascribed to a decrease in the presence of mitochondrial binding sites in altered livers [20]. This phenomenon, highlighted by the NAD(P)H_bound/free_ ratio values, a parameter that reflects the real involvement of the coenzymes in redox metabolism, is consistent with a decreased engagement of MCD livers in aerobic respiration, as supported by biochemical data on the decrease in ATP tissue content.

## 5. Conclusions

The MCD model of “fibrosing steatohepatitis” replicates the histologic features of human steatohepatitis and the sequence of steatosis, inflammatory cell injury, and fibrogenesis. As expected, the temporal sequence is consistent with a concept for involvement of oxidative injury in inflammatory recruitment and pathogenesis of hepatic fibrogenesis [31]. The AF results, supported by histochemical and biochemical assay data, confirm the potential of AF-OB analysis to provide a diagnostic response for a comprehensive and simultaneous detection and monitoring of fatty liver oxidative stress damages and disease progression. A basis is thus provided for possible applications in the biomedical research, such as in pharmacology to assess drug response and toxicity and in real-time diagnosis of organ functional alterations for the prescreening of donor organs in transplantation.
